# An Alternative Strategy for Pan-acetyl-lysine Antibody Generation

**DOI:** 10.1371/journal.pone.0162528

**Published:** 2016-09-08

**Authors:** Sun-Yee Kim, Choon Kiat Sim, Qiongyi Zhang, Hui Tang, Reinhard Brunmeir, Hong Pan, Neerja Karnani, Weiping Han, Kangling Zhang, Feng Xu

**Affiliations:** 1 Singapore Institute for Clinical Sciences, Agency for Science, Technology and Research, Singapore, Republic of Singapore; 2 Department of Pharmacology and Toxicology, University of Texas Medical Branch, Galveston, Texas, United States of America; 3 Laboratory of Metabolic Medicine, Singapore Bioimaging Consortium, Agency for Science, Technology and Research, Singapore, Republic of Singapore; 4 Department of Biochemistry, Yong Loo Lin School of Medicine, National University of Singapore, Singapore, Republic of Singapore; 5 Cardiovascular and Metabolic Disorders Program, Duke-NUS Graduate Medical School, Singapore, Republic of Singapore; Walter and Eliza Hall Institute of Medical Research, AUSTRALIA

## Abstract

Lysine acetylation is an important post-translational modification in cell signaling. In acetylome studies, a high-quality pan-acetyl-lysine antibody is key to successful enrichment of acetylated peptides for subsequent mass spectrometry analysis. Here we show an alternative method to generate polyclonal pan-acetyl-lysine antibodies using a synthesized random library of acetylated peptides as the antigen. Our antibodies are tested to be specific for acetyl-lysine peptides/proteins via ELISA and dot blot. When pooled, five of our antibodies show broad reactivity to acetyl-lysine peptides, complementing a commercial antibody in terms of peptide coverage. The consensus sequence of peptides bound by our antibody cocktail differs slightly from that of the commercial antibody. Lastly, our antibodies are tested in a proof-of-concept to analyze the acetylome of HEK293 cells. In total we identified 1557 acetylated peptides from 416 proteins. We thus demonstrated that our antibodies are well-qualified for acetylome studies and can complement existing commercial antibodies.

## Introduction

Lysine acetylation is a post-translational modification that has been extensively studied for their roles in cell signaling, transcription and metabolism through its effects on protein activity, subcellular localization and interaction with protein or DNA [1, 2]. Initial studies of acetylation have focused on single proteins such as tubulin, histones and p53, relying often on the use of radioactively labeled acetyl-CoA for selective acetylation of these proteins to study their biological functions [3–5]. Since the advent of proteomic approaches, scientists have advanced on studying entire acetylome. Various work have found more than a thousand acetylated proteins in the proteome, belonging to large macromolecular complexes involved in many cellular functions such as chromatin remodeling, nuclear transport and mitochondrial metabolism [6–8]. This has yielded tremendous progress in our understanding of the ubiquitous nature of this post-translational modification.

Because the nature of proteomic methods involves the obtaining of a large amount of information from a single sample, acetylome studies often require careful optimization to reduce the background [9]. To increase the true positives, the study of the acetylome by mass spectrometry typically requires a prior enrichment of acetyl-lysine peptides to remove non-acetylated peptides, particularly those that are highly abundant. Immunoprecipitation with pan-acetyl-lysine antibodies represents the most common method to enrich acetyl-lysine peptides [6]. It is often used in conjunction with trypsin, a protease that cleaves at unmodified lysine and arginine, thereby breaking large proteins into multiple smaller peptides [10]. The small molecular weight of the peptides and the exposure of the acetyl-lysine following tryptic cleavage enhance the immunoprecipitation of the peptides using a pan-acetyl-lysine antibody.

A key feature of acetyl-lysine antibodies that are suitable for acetylome studies is that they have high reactivity to a large number of acetyl-lysine substrates. One way to develop these antibodies is to use acetylated carrier proteins such as bovine serum albumin (BSA) as the antigen [11, 12]. The acetylation can be performed by a chemical reaction using acetic anhydride [13]. However, because there are only about 50 lysine residues on the BSA protein, the number of unique acetyl-lysine that can form antigens is limited. The use of such antibodies could restrict the number of acetylated proteins that can bind to them.

Here we report the use of a library of acetyl-lysine peptides as the antigen to generate pan-acetyl-lysine antibodies. These peptides consist of an acetyl-lysine in the middle of a random combination of ten amino acids, making the number of possible peptides reach about a trillion in theory. We have tested and verified by ELISA and dot blot that our antibodies are highly specific for acetyl-lysine peptides/proteins. We show that five of our antibodies, when pooled, perform complementary to, a commercial anti-acetyl-lysine antibody. The consensus sequences of the peptides bound by the two sets of antibodies are similar to a certain degree, suggesting that our approach is reasonable, yet they also differ moderately, which probably allows them to identify other acetylated proteins. Lastly, we used the antibodies to characterize the acetylome of HEK293 cells, a widely used cell line that nonetheless has not been well-studied for its acetylome, and identified 1557 acetylated peptides from 416 proteins in total.

## Materials and Methods

### Pan-acetyl-lysine Antibody Generation

For generation of pan-acetyl-lysine antibody, a random acetyl-lysine peptide library was synthesized (Mimotopes) and used as the antigen to immunize eight male New Zealand white rabbits (Cocalico Biologicals). The peptide sequence of the random library is NNNNNKacNNNNNC, in which N stands for an equimolar mixture of 18 amino acids with the exception of cysteine and methionine. To generate the antiserum, this random acetyl-lysine peptide library was conjugated to ovalbumin and injected to the rabbits. Three weeks after the initial immunization, a boost injection was performed. And the antiserum was collected one week after.

### Peptide Competition ELISA

The specificity of these pan-acetyl-lysine antibodies were tested by a peptide competition ELISA [14]. Briefly, the random acetyl-lysine peptide library was conjugated to keyhole limpet hemocyanin (KLH) as described by the manufacturer (Pierce) and coated to the microtiter plates at 4°C overnight. Subsequently, the lysine acetylated or unacetylated peptides was tested in the competition ELISA assay for their ability to inhibit the binding of the pan-acetyl-lysine antibody to random acetyl-lysine peptide-KLH conjugate. The antisera were used at 1:2000 dilution and the incubation was carried out for 2 hours at room temperature. The secondary goat anti-rabbit antibody was used at 1:1000 dilution and the incubation was performed at room temperature for 1 hour. Subsequently, an o-phenylenediamine (OPD) reaction (Pierce) was performed for color development. Finally, the ELISA plates were read for the absorbance at 492 nm on a plate reader (Tecan).

### Dot Blot Immunoassay

3T3-L1 cells treated with 10 μM EX527 (an inhibitor of deacetylase SIRT1) or 1 μM of Trichostatin A (TSA, Wako) and 10 mM of Nicotinamide (NAM, Sigma) were lysed in ETN buffer (1 mM EDTA, 100 mM NaCl, 50 mM Tris-HCl [pH = 8.0]) containing protease inhibitor cocktail. Cell lysates were sonicated and cleared by centrifugation at 12,000 rpm for 20 min at 4°C. Random peptides containing acetyl-lysine or non-acetyl-lysine were dissolved in 2 mM NaOH solution. The dot-blot apparatus (Biorad) was assembled with a 0.2 μm pore size nitrocellulose membrane that was pre-wetted into TBS then adjust the flow valve to open vacuum chamber. Samples were applied onto wells and allow the entire sample to filter through the membrane by gravity flow. The membrane was removed and rinsed with TBS. Immunoassay was conducted following manufacturer’s instruction and using specific antibodies against pan-acetyl-lysine (ICP0380, ImmuneChem), or SICS antisera from #37 to #44, respectively.

### Antibody Specificity Test against other Lysine Modifications

To test the specificity of these pan-acetyl-lysine antibodies against other forms of lysine modification, we synthesized three more random lysine modified peptide libraries (Mimotopes). The peptide sequence of these random libraries is the same as acetyl-lysine peptide library but the acetyl-lysine was replaced by di-methyl-lysine, propionyl-lysine and butyryl-lysine, respectively. Dot blot immunoassay was performed as described above using commercial pan-acetyl-lysine antibody (ICP0380), or SICS antisera from #38 to #42 against random lysine un-modified and modified peptide libraries. In ELISA assay, the random un-modified-lysine, di-methyl-lysine, propionyl-lysine and butyryl-lysine peptide libraries were conjugated to KLH and coated to the microtiter plates together with the random acetyl-lysine peptide-KLH conjugate at 4°C overnight. On the following day, the commercial pan-acetyl-lysine antibody (ICP0380), or SICS antisera from #38 to #42 were tested against random lysine un-modified and modified peptide-KLH conjugates as detailed above.

### Immunoprecipitation and Immunoblotting

HEK293 cells treated with TSA and NAM were lysed in RIPA lysis buffer (50 mM Tris-HCl [pH = 7.4], 150 mM NaCl, 0.1% SDS, 0.25% Na-deoxycholate, 1% NP-40) containing protease inhibitor cocktail (Roche). Immunoprecipitation was performed using either a control rabbit IgG or SICS antisera from #38 to #42 as described previously [15]. The immunoprecipitated proteins were then immunoblotted with a mouse pan-acetyl-lysine antibody (#9681, Cell Signaling).

### Preparing Cell Lysate and In-Solution Tryptic Digestion

To prepare proteins for mass spectrometry analysis, HEK293 cells treated with 10 mM of NAM and 1 μM of TSA were lysed in RIPA lysis buffer containing protease inhibitor cocktail (Roche). 20 mg of cell lysates were precipitated with Trichloroacetic acid (Sigma) and Acetone (Merck) at -20°C for 12 hours then pellets were resuspended in 100 mM NH4HCO3 [pH = 8.5] buffer. Samples were treated with sequencing-grade trypsin (V5111, Promega) at an enzyme/substrate ratio of 1:50 at 37°C for 16 hours. To ensure complete digestion, another batch of trypsin was added at an enzyme/substrate ratio of 1:100 and incubated for additional 4 hours at 37°C. Digested peptides were reduced, alkylated and quenched by DTT, iodoacetamide and cysteine, respectively. Trypsinized peptides were dried for 30 min at 30°C in a speedvac. The acK-peptide standard was prepared in parallel using acetylated BSA (ICP6090, ImmuneChem).

### Immunoprecipitation with Pan-acetyl-lysine Antibody

Pan-acetyl-lysine antibody (ICP0380, Immunechem) or mixture of SICS pan-acetyl-lysine antisera was added to digested peptides resolubilized in NETN buffer [16] and incubated at 4°C overnight with inverting. Dynabeads protein A was added to capture IgG and incubated with gentle mixing for 1 h at 4°C. The acetylated peptides bound to Dynabeads were washed four times with ETN buffer (50 mM Tris-HCl [pH = 8.0], 1 mM EDTA, 100 mM NaCl) using magnet, then eluted by washing three times with 1% TFA then completely dried. 100 fmol of trypsinized acetylated BSA peptides were added for internal normalization.

### LC-MS/MS Analysis of Immunoprecipitated Peptides

Peptide samples pulled down with the commercial pan-acetyl-lysine antibody or pool of SICS pan-acetyl-lysine antibodies were desalted, eluted, vacuum-dried, reconstituted, and then subjected to LC-MS/MS analysis as described previously [16].

### Peptide Identification from LC-MS/MS Data

Peptide identification was performed using the SEQUEST HT version 1.4 (Thermo Scientific) search engine and the human database was searched as described previously [16].

### Acetyl-lysine Peptide Sequence Motif Analysis

IceLogo was used to determine the peptide sequence motif around the acetyl-lysine [17]. A parameter of 5 amino acids was set around the acetylated lysines. In event that two acetylated lysine appear on a single peptide, the first acetylated lysine was used for the motif. P-value was set as 0.05. Fold change refers to the enrichment of the peptide motif above the pre-loaded human background proteome. The sequence motif was generated for each commercial antibody or our five SICS antibodies combined in a single mixture. A heatmap was also generated to show the enrichment of the various amino acids at each motif position.

### Gene Ontology Analysis

The classification of acetylated proteins according to their subcellular localization and molecular function was performed based on the information obtained from the UniprotKB/Swiss-Prot database. Gene ontology analysis of the acetylated proteins identified by either the commercial or the SICS pan-acetyl-lysine antibodies was carried out using Database for Annotation, Visualization, and Integrated Discovery (DAVID) [18].

### Statistics

For all statistics, a two-tailed student’s *t* test was performed. A p-value <0.05 was considered significant.

## Results

### Experimental Strategy to Generate and Validate Pan-acetyl-lysine Antibodies

**Fig 1:**
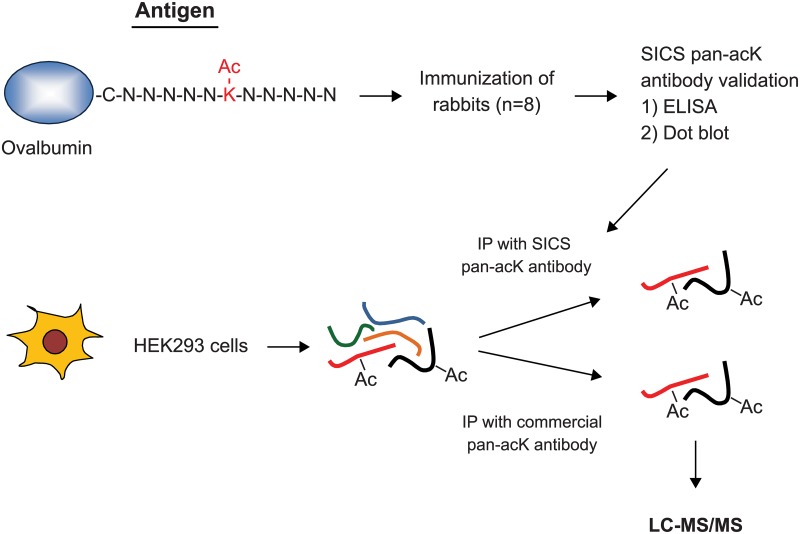
Strategy for pan-acetyl-lysine antibody generation and application in acetylome studies. To generate pan-acetyl-lysine (acK) antibody, a random acK peptide library conjugated to ovalbumin was used as an antigen to immunize rabbits. Raised antibodies were tested for specificity with ELISA and dot blot, and those that passed quality control were used to pull down acK peptides from HEK293 cell lysate for analysis by LC-MS/MS. A commercial pan-acK antibody was used for comparison.

To improve the coverage of acetyl-lysine peptides that are identified in acetylome studies, it is important to develop acetyl-lysine antibodies that are more diverse in their epitope recognition. We synthesized a library of acetyl-lysine peptides containing a random sequence NNNNNKacNNNNNC where N is any amino acid except cysteine and methionine (Fig 1). These peptides were conjugated to ovalbumin through the C-terminal cysteine and injected into rabbits to raise polyclonal antibodies. These antibodies were tested for sensitivity and specificity of binding to acetyl-lysines using peptide competition ELISA and dot blot. Antibodies that showed good reactivity and specificity were used to immunoprecipitate acetyl-lysine peptides in HEK293 cell lysate and the enriched acetylated peptides were identified by liquid chromatography-mass spectrometry.

### Validation of Pan-acetyl-lysine Antibodies by ELISA and Dot Blot

**Fig 2:**
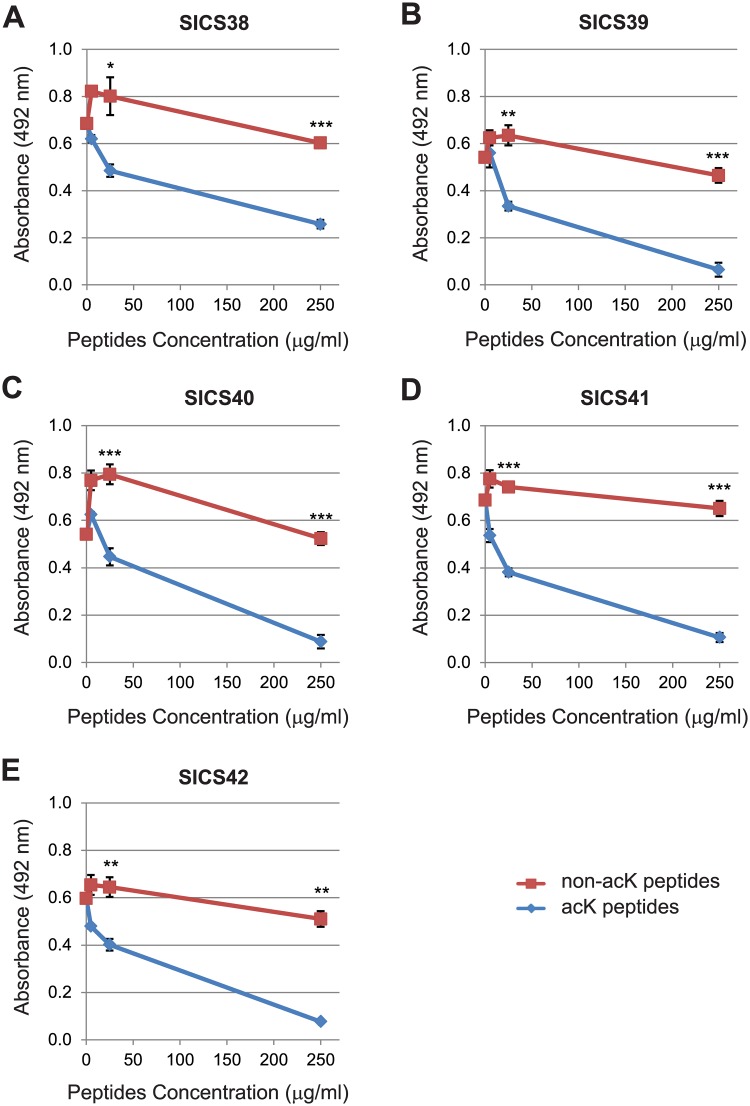
Validation of SICS pan-acK antibodies by peptide competition ELISA assay. A peptide competition ELISA assay was performed on each SICS pan-acK antibody (A to E) using a library of random non-acK peptides or acK peptides. The amount of competition is measured by the reduction of absorbance at OD 492 nm. Data are the averages of three independent measurements. * *P* <0.05, ** *P* <0.01, *** *P* <0.001 (Student’s *t*-test).

The specificity of our raised pan-acetyl-lysine antibodies was tested by a peptide competition ELISA assay and dot blot. The antibody is considered specific if both assays show a positive result. In the ELISA assay, the library of acetylated peptides was first conjugated to a carrier protein KLH and then coated to the microtiter plates. Then the acetyl-lysine antibodies were added together with either a library of competing acetylated or non-acetylated peptides. If the binding between acetyl-lysine antibodies and the immobilized acetylated peptides is weakened from the competing peptides, there will be less antibodies remain in the well after extensive washing which leads to decreased absorbance as examined by a plate reader at 492 nm wavelength. The antibody is considered specific for acetyl-lysines if the absorbance is reduced by the acetylated peptides but not by the non-acetylated peptides. We found that this is true for five of our antibodies, SICS38 to SICS42, as seen by the significantly larger drop of absorbance in the presence of competing acetylated peptides relative to non-acetylated peptides (Fig 2A to 2E).

**Fig 3:**
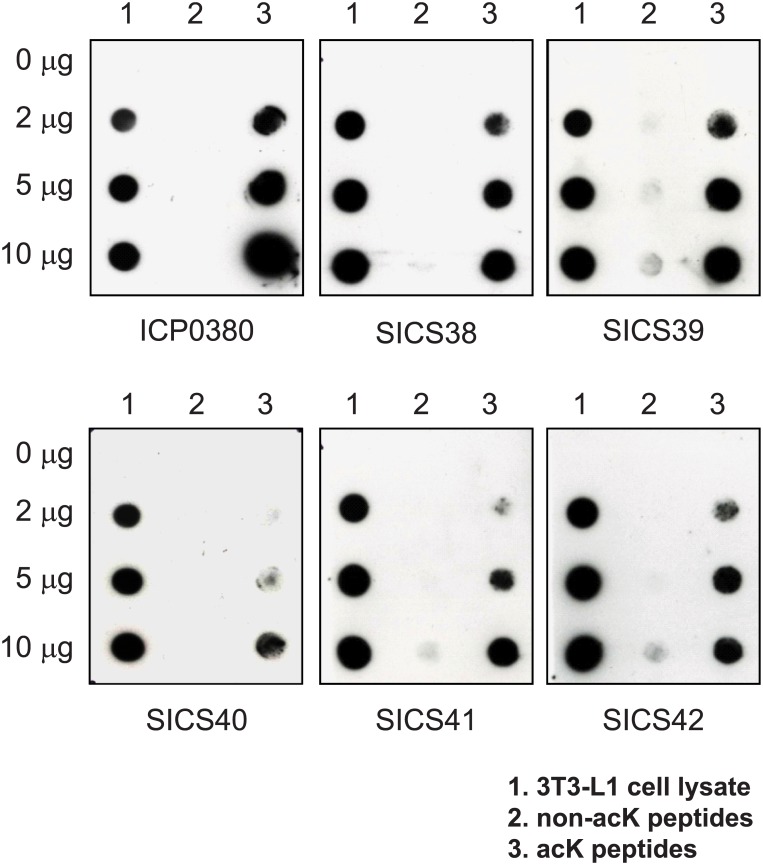
Validation of pan-acK antibodies by dot blot immunoassay. SICS pan-acK antibodies were tested by a dot blot immunoassay using: (1) 3T3-L1 cell lysate, (2) random peptides without acK, or (3) random peptides containing acK. 0, 2, 5, 10 μg of peptides or cell lysates were placed onto nitrocellulose membrane then detected by commercial pan-acK antibody ICP0380 or SICS pan-acK antibodies.

As a second test for the specificity of the antibodies, we conducted a dot blot where we tested each antibody against a library of non-acetylated peptides and a library of acetylated peptides. For positive control of acetylated proteins, we used lysate from EX527 treated 3T3-L1 cell as it is expected that the inhibition of a deacetylase SIRT1 will yield more acetylated proteins [19]. The rationale for the assay is that a specific antibody should recognize acetylated peptides and the 3T3-L1 lysate control but not the non-acetylated peptides. Indeed we found that similar to the commercial antibody ICP0380, five of our antibodies SICS38 to SICS42 reacted with the 3T3-L1 lysate and acetylated peptides and much less to the wild-type peptides (Fig 3). The intensity of binding increased with higher amount of 3T3-L1 cell lysate and acetylated peptides, suggesting that the binding is specific. Through ELISA and dot blot assays, we have shown that these five antibodies are compatible with acetylome studies. On the contrary, three other antibodies SICS37, SICS43 and SICS44 failed the ELISA test (S1 Fig) or the dot blot test (S2 Fig) and are excluded from the subsequent acetylome studies. Moreover, our pan-acetyl-lysine antibodies SICS38 to SICS42 could also clearly detect the increase in the level of global lysine acetylation in 3T3-L1 cells treated with the lysine deacetylase inhibitors Trichostatin A and Nicotinamide (S3 Fig), further demonstrating their specificity against acetylated lysine.

**Fig 4:**
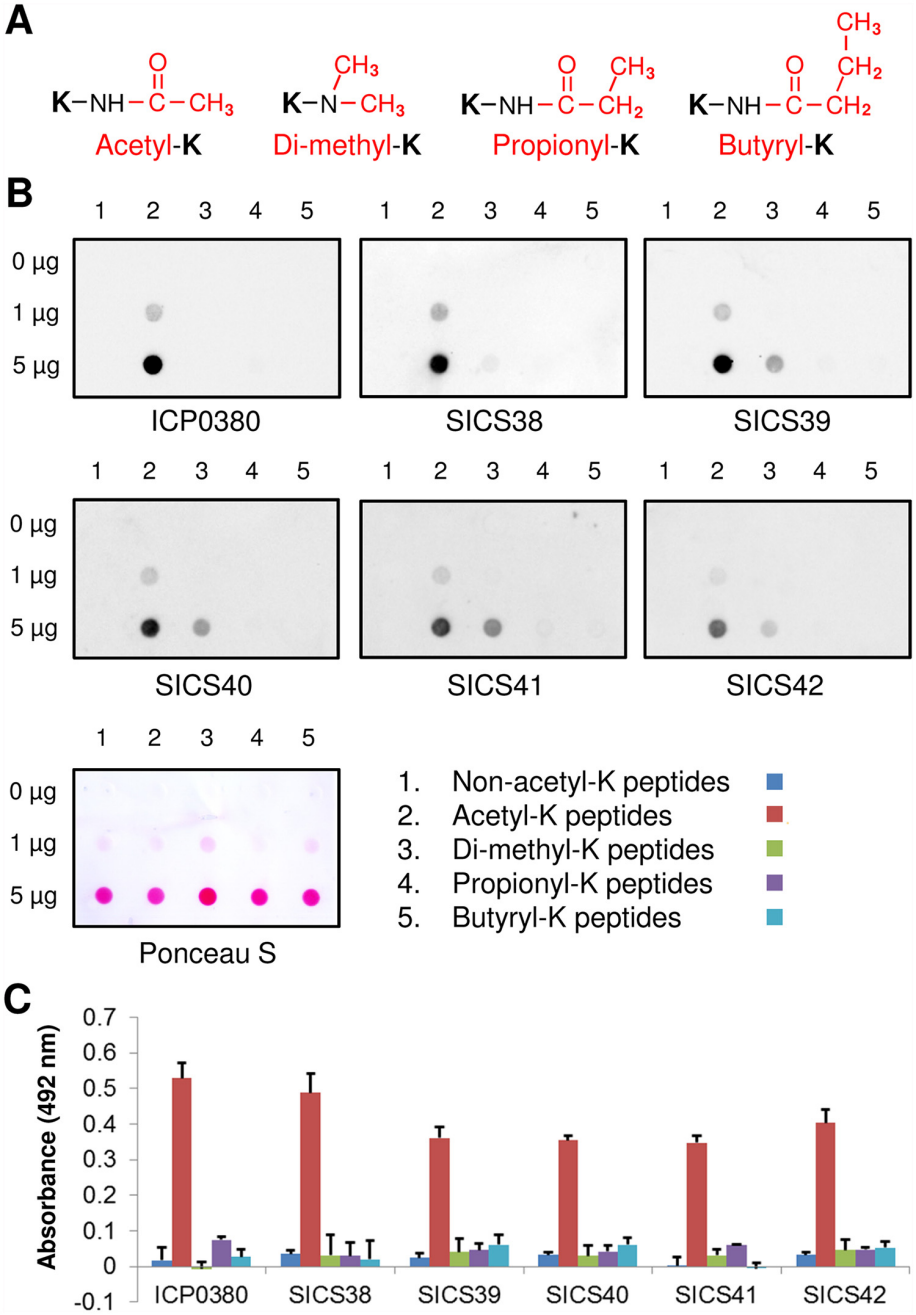
Validation of pan-acK antibodies against other lysine modifications. (A) Chemical structures of acetyl-, di-methyl-, propionyl- and butyryl-lysine. Post-translational modifications were highlighted in red. (B) SICS pan-acK antibodies were tested by a dot blot immunoassay against: (1) wild-type peptides, (2) acetyl-lysine peptides, (3) di-methyl-lysine peptides, (4) propionyl-lysine peptides, and (5) butyryl-lysine peptides. 0, 1, 5 μg of peptides were spotted onto nitrocellulose membrane then detected by commercial pan-acK antibody ICP0380 or SICS pan-acK antibodies. (C) SICS pan-acK antibodies #38 to #42 and the commercial pan-acK antibody ICP0380 were tested by ELISA assay against various lysine modified and un-modified peptide-KLH conjugates.

To further test the specificity of our pan-acetyl-lysine antibodies SICS38 to SICS42 against other forms of lysine modification, we selected lysine di-methylation, propionylation and butyrylation, whose chemical structures are similar to lysine acetylation (Fig 4A), and synthesized these random lysine modified peptide libraries. In dot blot immunoassays, we found that our antibodies SICS38 to SICS42 reacted strongly with acetylated peptides, and showed a slight cross-reactivity with di-methylated peptides, but did not react with the wild-type, propionylated or butyrylated peptides at a detectable level (Fig 4B). In addition, using ELISA assay described in [20], we observed much stronger reactivity of our antibodies SICS38 to SICS42 against acetyl-lysine peptide-KLH conjugate than the un-modified-lysine, di-methyl-lysine, propionyl-lysine and butyryl-lysine peptide-KLH conjugates (Fig 4C).

Finally, to examine whether our pan-acetyl-lysine antibodies could actually pull-down acetylated proteins from the whole cell lysates, we performed immunoprecipitation assay [15] using either a control rabbit IgG or our antibodies SICS38 to SICS42. As shown in S4 Fig, all five SICS antibodies could immunoprecipitate significant amount of acetylated proteins from the whole cell lysates of TSA and NAM treated HEK293 cells as detected by immunoblotting. In summary, we showed that our pan-acetyl-lysine antibodies SICS38 to SICS42 strongly recognize lysine acetylation thus are compatible with acetylome studies.

### Comparison of Our Pan-acetyl-lysine Antibodies with a Commercial Antibody

**Fig 5:**
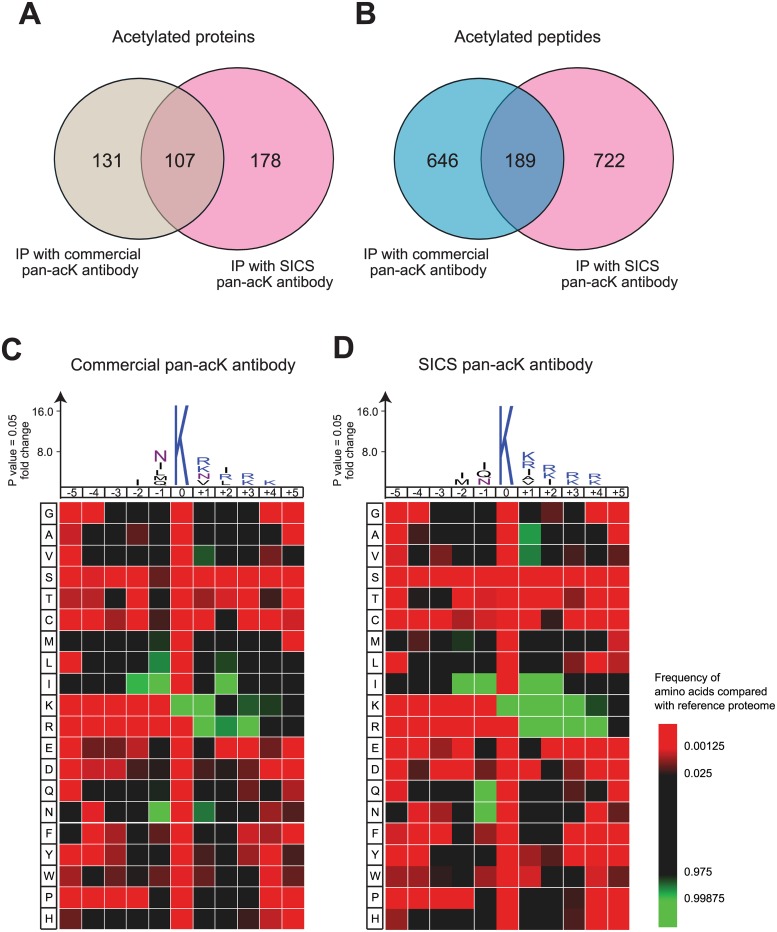
Identification of acetylated peptides by LC-MS/MS and their consensus sequence motif. Overlap of identified acetylated proteins (A) and peptides (B) from HEK293 cells using commercial pan-acK antibody or SICS pan-acK antibodies. (C and D) Peptide sequence motif and heat map of amino acids flanking the acetyl-lysine (*P* < 0.05). The height of amino acids on the peptide motif represents fold change above background. Significance was set at *p*-value < 0.05. The color intensity of an amino acid in the heatmap reflects the difference in the frequency of an amino acid in the experimental data set and its reference data set.

We proceeded to pool the five SICS antibodies in a single cocktail and used it to perform an acetylome study on HEK293 cells. The rationale for pooling the antibodies is that if each antibody binds a certain range of acetyl-lysine substrates based on flanking sequences around the acetyl-lysine, pooling will have a certain additive effect on the total range of substrates that can be bound. A previously published paper showed that only a small number of about 30 unique acetylated peptides can be identified in HEK293 cells [21]. In our study, the pooling of five SICS pan-acetyl-lysine antibodies found a much larger number of acetylated peptides—a total of 911 peptides that represented 285 proteins (Fig 5A and 5B). These numbers are also slightly higher than those found by a commercial antibody in a parallel experiment (835 peptides from 238 proteins). We have thus uncovered a larger number of acetyl-lysine proteins in the HEK293 cells. Notably, 107 proteins are shared between the commercial and SICS antibodies, suggesting that our antibodies bound to a common, as well as a complementary pool of substrates to the commercial acetyl-lysine antibody widely used in acetylome studies. The entire list of acetylated peptides is found in S1 Table.

To test how similar are the substrates bound by the commercial versus SICS antibodies, we aligned the peptides around the acetyl-lysines and generated a peptide motif to find the most common amino acids flanking the acetyl-lysines. A higher fold change of amino acid above the proteome suggests that that amino acid is more frequently acetylated compared with background levels. The results surprisingly show a similar pattern of peptide motif between commercial and SICS antibodies. Particularly, the amino acids that lie at the -2 and -1 positions contain isoleucine (I), methionine (M), asparagine (N), glutamine (Q), leucine (L) for the commercial antibody and a very similar isoleucine (I), methionine (M), asparagine (N), glutamine (Q) for the SICS antibodies (Fig 5C and 5D). At the +1 and +2 positions from the acetyl-lysine, there are valine (V), isoleucine (I), asparagine (N), leucine (L) for the commercial antibody and a similar valine (V), isoleucine (I), alanine (A) for the SICS antibodies. The differences at the +1, +2 positions might explain the non-overlapping set of proteins found in the acetylome from the two classes of antibodies. Notably, we also found an enrichment of lysine (K) or arginine (R) at +1/+2/+3/+4 positions and we observed that the vast majority of them are unacetylated and cause termination of the peptides through tryptic cleavage. To show a detailed representation of the frequency of each amino acid at each position in the peptide motif, a heatmap is also constructed. This gives a view of also the amino acids that are at levels below the proteomic baseline (i.e. in red), and there are similarities of underrepresentation between the commercial and SICS pan-acetyl-lysine antibodies, at amino acids serine (S), threonine (T), cysteine (C), glutamic acid (E), with varying differences of the other amino acids (Fig 5C and 5D).

### Characterizing the Acetylome of HEK293 Cells

**Fig 6:**
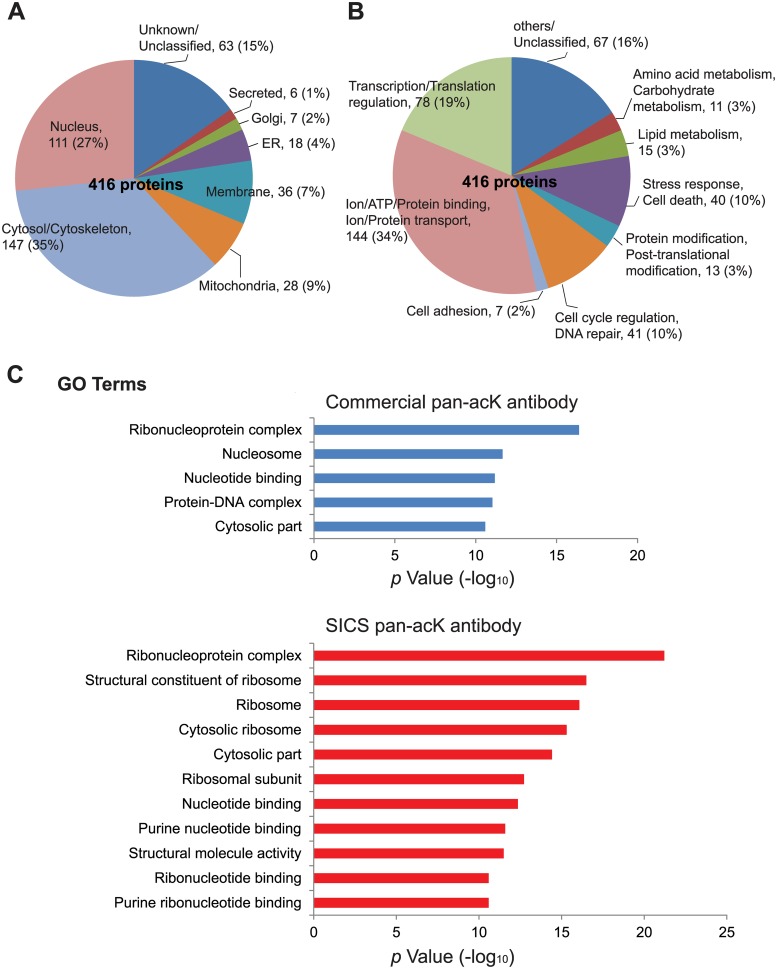
Classification and gene ontology of acetylated proteins. Acetylated proteins identified by LC-MS/MS after immunoprecipitation with both the commercial and SICS pan-acK antibodies were classified by their subcellular localization (A) and molecular function (B). (C) The most significant (*p* value (-log_10_) > 10) GO terms for the acetylated proteins identified by either the commercial or the SICS pan-acK antibodies.

As our study identified about 50 times more acetyl-lysine peptides than the previous study [21] and could be a useful resource for other scientists, we categorized the acetylome of HEK293 cells according to their subcellular localization and molecular function. Out of the entire pool of 416 proteins enriched by the commercial and homemade antibodies, we found that 35% of them are in the cytosol, 27% in the nucleus and 9% in the mitochondria (Fig 6A). As for molecular function, 34% of the proteins are involved in the binding or transportation of ion or proteins, 19% in transcription or translation, 10% in stress response, and 10% in cell cycle/DNA repair (Fig 6B). Moreover, to investigate whether the acetylated proteins identified by either the commercial or the homemade pan-acetyl-lysine antibodies associate with any specific cellular functions, we performed Gene Ontology (GO) enrichment analysis using the online bioinformatics tool DAVID [18]. We found that, in addition to some of the GO terms shared by both the commercial and SICS antibodies, such as “ribonucleoprotein complex”, “nucleotide binding”, and “cytosolic part”, both antibodies immunoprecipitate their unique sets of acetylated proteins. For example, acetylated proteins involved in “nucleosome” and “protein-DNA complex” were specifically enriched by the commercial antibody, while acetylated proteins involved in “structural constituent of ribosome”, “ribosome” and “structural molecule activity” were specifically immunoprecipitated by the SICS antibodies (Fig 6C). A detailed list of the GO terms for the acetylated proteins identified by both the commercial and SICS antibodies can be found in S2 Table.

## Discussion

Current approaches to characterize the lysine acetylome involve mass spectrometry following the enrichment of acetylated peptides using anti-acetyl-lysine immunoprecipitation. The success of such experiment depends crucially on the affinity of the antibody towards acetyl-lysine peptides; it has to be sensitive enough to accommodate for structural and charge variances found in different amino acid sequences surrounding the acetyl-lysine, but also specific enough to react only to acetyl-lysines and not closely related residues such as propionyl-lysines [22]. While some studies prefer antibodies that could distinguish between closely related acetyl-lysine residues such as the K9 and K27 on histone H3, acetylome studies favor antibodies that are undiscriminating towards any particular acetyl-lysines.

Here we have shown that using a random library of antigen peptides to generate the antibody can provide complementary coverage of binding the acetylated proteins in the proteome to a commercial pan-acK antibody generated using acetylated KLH as the antigen. We have found that when characterizing the acetylome of HEK293 cells, our pool of five polyclonal antibodies bind almost 300 acetylated proteins, among which nearly 200 proteins (possibly some of them are novel) are not identified by the commercial antibody. Acetylated proteins include not only the abundant histones and tubulin but also the more rare proteins such as zinc finger protein 12 found in the nucleus.

A recent paper suggested that combining multiple clones of antibodies can broaden the range of permissible binding of acetylated proteins [20]. In a similar way, we pooled antibodies to form a master antibody cocktail and used it to enrich acetylated peptides. A minor difference is that while they have combined monoclonal antibodies, we have pooled polyclonal antibodies. In our acetylome study, we have enriched nearly three hundred acetylated proteins in the HEK293 proteome and this number is much fewer than the Svinkina *et al*. study. This difference is likely due to the sensitivity of mass spectrometry instrument [16, 20] and the methodologies used in these two acetylome studies. For example, we performed LC-MS/MS analysis without pre-fractioning the immunoprecipitated acetylated peptides, while the Svinkina *et al*. study used a stable isotope labeling method for quantitative analysis of acetylated peptides. It is likely that further enhancement of our protocols and mass spectrometry methodologies can improve the results.

We have used whole cell lysate in our characterization of HEK293 acetylome. Although the cell line has been extensively used for molecular biology, its acetylome has not been studied in detail. A recent acetylome study has found only 30 acetylated peptides in HEK293 cells [21], and in this light, our work has provided a significant improvement in coverage showing 1557 acetylated peptides in total. Looking ahead, one area for further testing of the antibody would be to sub-fractionate the cellular components into nucleus, cytoplasm and mitochondria, as it has been shown that the sequence motif for acetyl-lysine sites can be distinctly different between subcellular locations [23]. Although the sequence motif published in this paper differs from the sequence motif of the commercial antibody, we note that a different antibody has been used, and as seen in Svinkina *et al*. [20], the motif depends critically on the nature of the antibody used. Moreover, as sequence motifs are known to be well-conserved across species, for example, between Drosophila and humans [24], our antibodies can be tested in different species to show its compatibility to acetylated proteins in multiple species. We believe that our antibody will be a useful tool to complement the use of commercial antibodies in the study of acetylomes, offering further insights into cell signaling, transcription and metabolism.

## Supporting Information

S1 Fig SICS pan-acK antibodies that did not pass quality control of peptide competition ELISA assay. A peptide competition ELISA assay was performed on each SICS pan-acK antibody (A to C) using a library of random non-acK peptides or acK peptides. The amount of competition is measured by the reduction of absorbance at OD 492 nm. Data are the averages of three independent measurements. * *P* <0.05, ** *P* <0.01, *** *P* <0.001 (Student’s *t*-test).
(EPS)

S2 Fig SICS pan-acK antibodies that did not pass quality control of dot blot immunoassay. SICS pan-acK antibodies were tested by a dot blot immunoassay using: (1) 3T3-L1 cell lysate, (2) random peptides without acK, or (3) random peptides containing acK. 0, 2, 5, 10 μg of peptides or cell lysate were placed onto nitrocellulose membrane then detected by SICS pan-acK antibodies #37, #43 and #44.
(EPS)

S3 Fig SICS pan-acK antibodies detected the increases in global lysine acetylation in 3T3-L1 cells treated with lysine deacetylase inhibitors. SICS pan-acK antibodies were tested by a dot blot immunoassay using: (1) lysate from un-treated 3T3-L1 cells, (2) lysate from Trichostatin A and Nicotinamide treated 3T3-L1 cells, (3) random peptides without acK, or (4) random peptides containing acK. 1, 5, 10 μg of peptides or cell lysates were spotted onto nitrocellulose membrane then detected by commercial pan-acK antibody ICP0380 or SICS pan-acK antibodies #38 to #42.
(EPS)

S4 Fig SICS pan-acK antibodies immunoprecipitated acetylated proteins from HEK293 cell lysate. SICS pan-acK antibodies were tested in immunoprecipitation assay using lysates of TSA and NAM treated HEK293 cells. A rabbit IgG was included as a control. Immunoprecipitated acetylated proteins were detected by immunoblotting using a mouse pan-acK antibody.
(EPS)

S1 Table List of the acetylated peptides identified in the acetylome studies in HEK293 cells using commercial and homemade pan-acetyl-lysine antibodies. (XLS)

S2 Table Gene ontology analysis of the acetylated proteins identified in this acetylome study by commercial and SICS pan-acetyl-lysine antibodies. (XLS)
